# The effects of nicorandil on microvascular function in patients with ST segment elevation myocardial infarction undergoing primary PCI

**DOI:** 10.1186/s12947-015-0020-9

**Published:** 2015-05-27

**Authors:** Jelena Kostic, Ana Djordjevic-Dikic, Milan Dobric, Dejan Milasinovic, Milan Nedeljkovic, Sinisa Stojkovic, Jelena Stepanovic, Milorad Tesic, Zoran Trifunovic, Danijela Zamaklar-Tifunovic, Mina Radosavljevic-Radovanovic, Miodrag Ostojic, Branko Beleslin

**Affiliations:** Clinic for Cardiology, Clinical Center of Serbia, Visegradska 26, Belgrade, Serbia; Medical School, University of Belgrade, Belgrade, Serbia; Military Medical Academy, Belgrade, Serbia; Medical Faculty, University of Defense, Belgrade, Serbia

**Keywords:** STEMI, Primary PCI, Nicorandil, Microvascular dysfunction, Index of microvascular resistance, Transthoracic Doppler derived coronary flow reserve

## Abstract

**Background:**

Nicorandil, as a selective potassium channel opener, has dual action including coronary and peripheral vasodilatation and cardioprotective effect through ischemic preconditioning. Considering those characteristics, nicorandil was suggested to reduce the degree of microvascular dysfunction.

**Methods:**

Thirty-two patients with ST-elevation myocardial infarction undergoing primary percutaneous coronary intervention (pPCI) were included in the study. Index of microvascular resistance (IMR) was measured in all patients immediatelly after pPCI before the after administration of Nicorandil. ST segment resolution was monitored before intervention and 60 min after terminating the procedure. Echocardiographic evaluation of myocardial function and transthoracic Doppler derived Coronary flow reserve (CFR) of infarct related artery (IRA) was performed during hospitalization and 3 months later.

**Results:**

IMR was significantly lower after administration of Nicorandil (9.9 ± 3.7 vs. 14.1 ± 5.1, *p* < 0.001). There was significant difference in ST segment elevation before and after primary PCI with administration of Nicorandil (6.9 ± 3.7 mm vs. 1.6 ± 1.6 mm, *p* < 0.001). Transthoracic Doppler CFR measurement improved after 3 months (2.69 ± 0.38 vs. 2.92 ± 0.54, *p* = 0.021), as well as WMSI (1.14 ± 0.17 vs. 1.07 ± 0.09, *p* = 0.004).

**Conclusion:**

Intracoronary Nicorandil administration after primary PCI significantly decreases IMR, resulting in improved CFR and ventricular function in patients with STEMI undergoing primary PCI.

## Introduction

In patients with ST segment elevation myocardial infarction (STEMI) undergoing primary percutaneous coronary intervention (pPCI), despite early reperfusion and achievement of epicardial coronary artery patency, some patients still exhibit reduced or no coronary flow to distal microcirculation. This complication is not naïve, as it has been shown that patients with no-reflow phenomenon have worse myocardial function and prognosis in the long term follow-up [1, 2]. The mechanism of microcirculatory dysfunction is multifactorial with vasospasm of microcirculation in the center of different pathophysiological mechanisms [3]. Thus, several treatment options including nitroprusside, adenosine and verapamile, that all induce vasodilatation have been proposed to deal with reduced or no coronary flow [4–8].

Nicorandil is a nicotinamide ester with the dual mechanism of action, combining activation of ATP-sensitive potassium (K-ATP) channels with nitro-vasodilator (nitric oxide donor) actions. By selective activation of the K-ATP channels at the sacrolemmal and mitochondrial level, nicorandil induces coronary and peripheral vasodilatation, with subsequent reduction of preload and afterload. The role of the K-ATP channels in ischemic preconditioning suggests that nicorandil has cardioprotective effect [9]. Considering those mechanisms of action, it has been shown previously that nicorandil might have an important role in the treatment of no re-flow following pPCI in acute myocardial infarction [10]. In fact, nicorandil was suggested to reduce the degree of microvascular dysfunction [11, 12].

The aim of this study was to evaluate the effects of nicorandil on microcirculatory function as evaluated by the index of microcirculatory resistance. In addition, it was of particular clinical importance to evaluate the effects of nicorandil on the improvement of ECG ST segment changes, coronary flow reserve (CFR) and myocardial function by evaluating wall motion score index (WMSI).

## Methods

### Study population

The study was designed as a single center prospective study. Thirty-two patients (mean age 56.5 ± 9.0 years, male 26, and female 6 patients) with ST segment elevation myocardial infarction (STEMI) were enrolled in the study. All patients were treated according to current recommendations for STEMI treatment [13], and Nicorandil was administered in a “top-of-the-treatment” manner after primary PCI. The diagnosis of STEMI was made according to chest pain lasting for more than 20 min and ST segment elevation of more than 2 mm in at least two contiguous leads, further confirmed by an increase in cardiospecific enzymes. Immediately after diagnosis was made, the patients were referred to pPCI with symptoms of STEMI of less than 12 h of duration, and pretreated before arrival to catheterization laboratory with clopidogrel 600 mg and aspirin 300 mg. The study was approved by ethical committee of Medical faculty, University of Belgrade, Belgrade, Serbia.

### Study protocol

All patients presented to catheterization laboratory as Killip Class I or II. Before starting pPCI of the culprit artery, all patients received intravenous heparin (70 IU/kg body mass). PPCI was performed in the usual manner. Balloon predilatation, thromboaspiration (6 or 7 F Export aspiration catheter, Medtronic, USA) as well as administration of glycoprotein IIb/IIIa inhibitors were performed or administered when needed according to operator’s decision.

Measurements of index of microcirculatory resistance (IMR) were performed after successful PCI by the Radianalyzer™Xpress measurement system (St. Jude Medical, Minneapolis, Minnesota) with the pressure wire (Certus, St. Jude Medical, USA) positioned in the distal part of the culprit coronary artery. After measurement of the resting transit times with injection of 3–4 mL of saline three times intracoronary, maximal hyperemia was induced by intracoronary bolus of papaverine (15 mg for left coronary artery or 10 mg for right coronary artery), and hyperemic mean transit times were obtained by repeated injections of intracoronary saline for three times (3–4 mL per time). Then, 12 mg of Nicorandil was administered intracoronary, and the same IMR measurements were repeated 10 min later. IMR was calculated by multiplying distal pressure by hyperemic mean transit time.

ECG ST segment elevation [14] was analyzed at baseline (before primary PCI) and 60 min after the end of the procedure, and the difference between basal and post PCI ST segment elevation was calculated. Thrombolysis in Myocardial Infarction (TIMI) flow grade [15] and Myocardial Blush Grade (MBG) [16] were analyzed before pPCI in all patients, and after Nicorandil administration. Two cardiologists who were not aware of the clinical data of the patients evaluated ST-elevation, TIMI and MBG score.

### Evaluation of global left ventricular function and coronary flow reserve

Transthoracic echocardiogram (Acuson Sequoia C256, Siemens Medical Solutions USA) was performed on the day after pPCI with evaluation of myocardial function and coronary flow reserve. A 17-segment model was used to determine systolic left ventricular (LV) function [17] by wall motion score index (WMSI) that was calculated by dividing the sum of individual visualized segment scores by the number of visualized segments.

Transthoracic Doppler echocardiographic examination was performed with the commercially available digital ultrasound system (Sequoia C256 Acuson Siemens Mountain View, California) with 3V2C mutifrequency transducer using second harmonic technology. After standard examination, distal left anterior descending artery or postero-descedant branch of right coronary artery was evaluated with 4 MHz transducer. For visualization of the flow in the coronary artery was used color Doppler matched Nyquist limit of 16–30 cm / s, while the flow rate is measured by pulsed Doppler. For the evaluation of flow in the distal part of the left anterior descending artery was used the modified apical three cavities with acoustic window in the level of midclavicular line in the fourth and fifth intercostals spaces in the left lateral decubitus position. For the assessment of coronary flow in the distal right coronary artery standard apical longitudinal view was used. From this position the probe is turned slightly anti-clockwise and leaned slightly forward, until a coronary flow in last interventricular sulcus using color Doppler is obtained. The sample pulse Doppler width of 5 mm is placed on the color signal of a coronary artery in its distal part. Spectral Doppler signal coronary artery shows a characteristic biphasic appearance with lower systolic and diastolic greater component. A sample volume (3–5 mm wide) was positioned on the color signal of the distal infarct related artery. Flow velocity recordings were performed with the stable transducer position at rest and maximal hyperemia, which was induced by administration of intravenous adenosine (140 mcg/kg over 2 min). CFR was obtained as the ratio of peak diastolic flow velocity during vasodilation and diastolic flow velocity at baseline [18, 19].

Follow-up echocardiographic examination with evaluation of WMSI and CFR was performed 3 months after pPCI.

### Statistical analysis

The sample size was calculated using the program G*Power. In a previous randomized study (QUOTE) [20], which compared the values of IMR in patients undergoing PCI in stable coronary artery disease who received placebo or Nicorandil. Considering difference between placebo and Nicorandil (IMR values were 25.4 ± 12.1, and 17.9 ± 9.1), α = 0.05 and the power of the statistical test (1-β) = 0.95, the needed sample size was estimated to include at least 28 patients.

The normality assumption for continuous variables was evaluated by the Kolmogorov­Smirnov test. Continuous variables are presented as means and standard deviations for normally distributed variables or as medians and interquartile ranges (IQR) for non-normal distribution. They were compared using Students *t*-test, or its non-parametric equivalent Mann–Whitney *U* test. Categorical variables were presented as counts and percentages and were compared with the chi-square or Fisher’s exact test. Relation of various numerical and dichotomous variables to IMR was evaluated using Pearson’s’ correlation. Univariable and multivariable linear regression (stewise model) was used to determine predictors of IMR and CFR. For all analysis, a two-sided *p* < 0.05 was considered statistically significant. All data were processed using the statistical package for social sciences, version (SPSS, Chicago, Ill).

## Results

Patients demographic and risk profile characteristics are presented in Table 1. Clinical, procedural and angiographic data are presented in Table 2. PPCI was angiographically successful in all patients with achievement of TIMI and MBG grade 3 in all patients. Total ischemic time was 225 (144 IQR) minutes; there were 13 patients with anterior wall STEMI, and 19 patients with inferior wall STEMI (13 LAD, 19 RCA). The peak values of cardiac markers were: CK 1778 ± 1108 U/l, CK MB 148 ± 116 ng/ml, and troponin 32 ± 46 ng/ml.

**Table 1:** Patients demographic characteristics

	Study group (*n* = 32)
Male gender, *n* (%)	26 (81.3 %)
Age (years)	56.5 ± 9.0
Hypertension, *n* (%)	16 (50 %)
Dyslipidemia, *n* (%)	13 (41 %)
Diabetes	
Oral therapy, *n* (%)	11 (34.4 %)
Insulin therapy, *n* (%)	1 (3 %)
Smokers	
Current, *n* (%)	16 (50 %)
Ex smokers, *n* (%)	5 (16 %)
Family history of CAD, *n* (%)	19 (59.4 %)
Body mass index (kg/m^2^)	28.8 ± 4.7

**Table 2:** Clinical, procedural and angiographic data

	Study group (*n* = 32)
Pain to door time, median (IQR) minutes)	161 (134)
Door to balloon time, median (IQR) (minutes)	64 (57)
Total ischemic time, median (IQR) (minutes)	225 (144)
Killip class on admission	
Class I	23 (72 %)
Class II	9 (28 %)
Anterior wall MI	13 (40 %)
Infarct related artery system	
LAD	13 (40 %)
RCA	19 (60 %)
TIMI flow grade 0 before	31 (97 %)
Manual thromboaspiration	7 (30 %)
Balloon predilatation	15 (47 %)
GP IIb/IIIa inhibitors	3 (10 %)
Number of stents implanted	1.38 ± 0.61
Total stent length (mm)	21.8 ± 4.8
Average stent diameter (mm)	3.14 ± 0.38
Balloon postdilatation	2 (6.3 %)
TIMI flow grade 3 after	32 (100 %)
Myocardial blush grade 3 after	32 (100 %)
Creatine kinase (U/L)	1778 ± 1108
Creatine kinase MB (U/L)	148 ± 116
Troponin (μg/L)	32.3 ± 45.9

There was a significant resolution of ST segment elevation after pPCI in comparison to hospital admission ECG (6.9 ± 4.0 mm vs. 1.6 ± 1.6 mm, *p*<0.001).

**Fig. 1:**
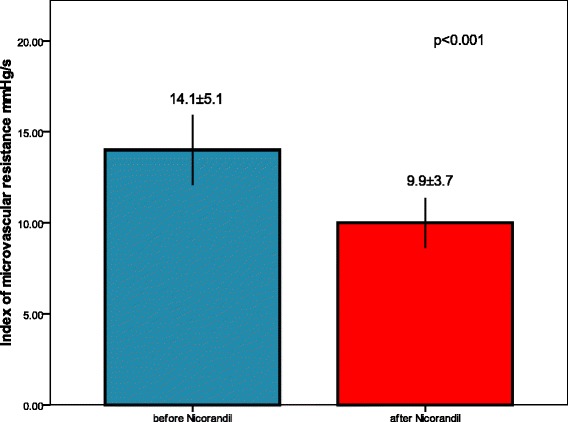
IMR in infarct related artery before and after Nicorandil administration

**Fig. 2:**
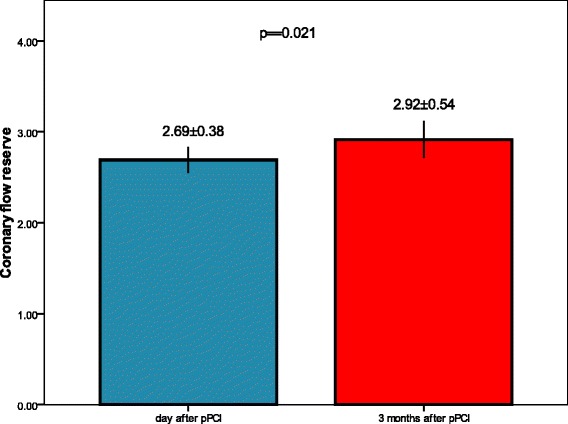
Transthoracic Doppler derived CFR day after and in follow-up

**Fig. 3:**
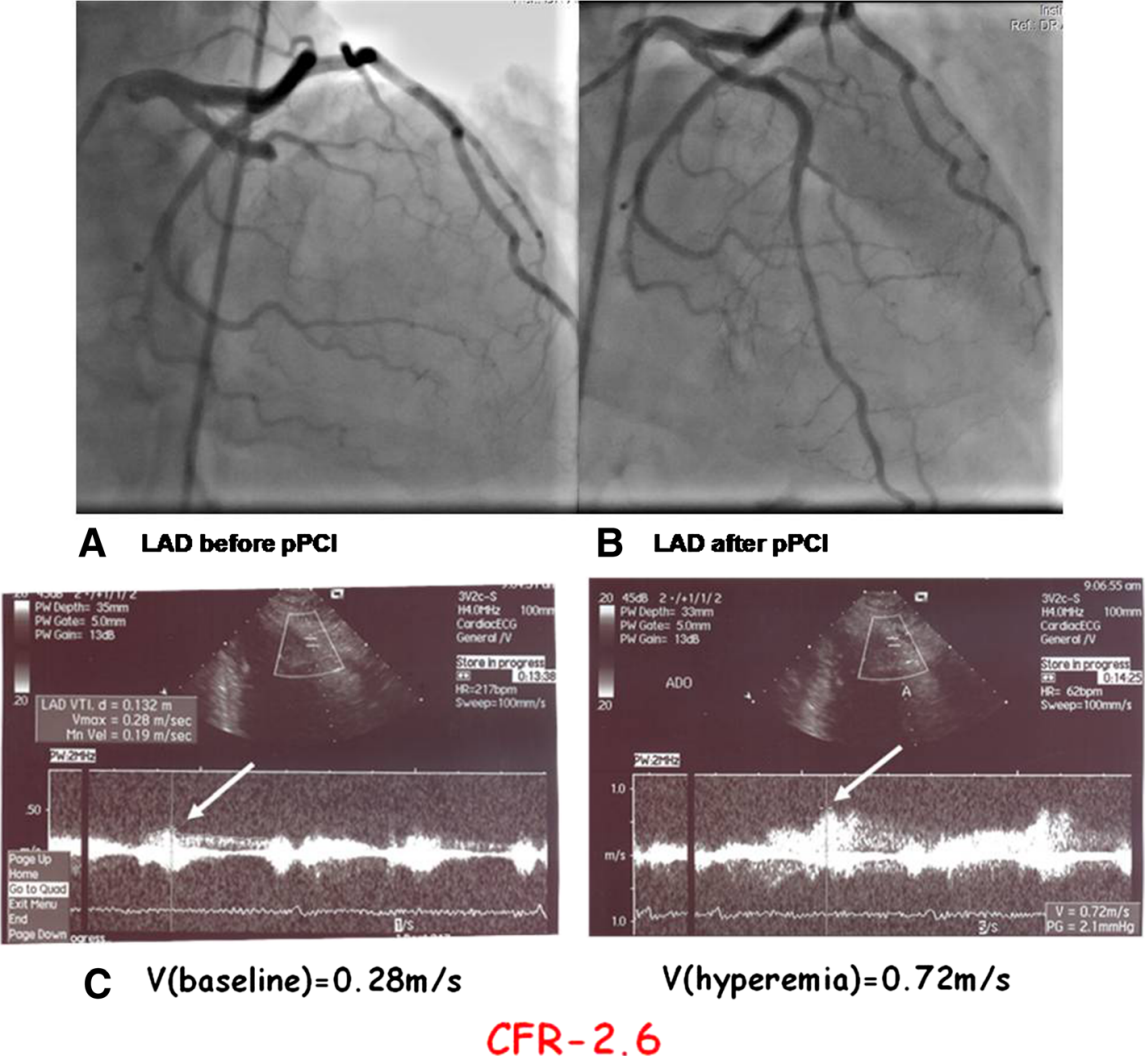
Patient with anterior AMI: **a** pPCI, **b** IMR after Nicorandil, **c** CFR in day after pPCI

IMR was statistically significantly lower after administration of Nicorandil in comparison to IMR before Nicorandil administration 14.1 ± 5.1 vs. 9.9 ± 3.7, *p* < 0.001 (Fig. 1). Transthoracic Doppler derived CFR (Fig. 2), performed the day after pPCI was 2.69 ± 0.38, and further significantly improved after 3 months to 2.92 ± 0.54, *p* = 0.021. WMSI measured one day after pPCI improved from 1.16 ± 0.17 to 1.07 ± 0.09, *p* = 0.004, after 3 months follow-up. Figure 3a, b and c demonstrate a patient with acute anterior myocardial infarction treated successfully with pPCI who after Nicorandil administration had IMR 8.69, CFR 2.6, and WMSI 1.12.

There was a correlation between IMR after Nicorandil administration and WMSI at 3 months follow-up period (*r* = 0.376, *p* = 0.034), as well as between IMR after nicorandil administration and noninvasive CFR the day after primary PCI (*r* = 0.510, *p* = 0.003) as well at 3 months follow-up period (*r* = 0.437, *p* = 0.012). Nicorandil administration was the only univariate predictor of the final IMR (*p* = 0.003). IMR after nicorandil administration was the only univariate predictor of nonivasive CFR the day after (*p* = 0.003) and in the 3 months follow-up period after primary PCI (*p* = 0.012).

During the follow-up period of average 1.5 year we had only one patients with non cardiac related death. There were no heart failures, re-infarction, re-hospitalisation, re-intervention or cardiac death.

## Discussion

The main findings of our study indicate that Nicorandil improves microvascular function, as presented by lower index of microvascular resistance and normal CFR immediately one day after pPCI that further improved after 3 months, which all translated into improved myocardial function as demonstrated by better WMSI.

Preservation of microvascular integrity, prevention of inflammatory damage and metabolic support of ischemic myocardium are important in the prevention and decrease of vascular-reperfusion injury [3]. The complex pathogenesis of microvascular injury: production of oxygen free radicals, neutrophil activation, endothelial and myocites swelling, loss of antioxidant enzymes and cardiomyocite apoptosis [3, 21], urges for new preventive solutions. In contrast to classic nitrates that have dilator effect on large conductive vessels (epicardial vessels), Nicorandil has greater effect on small resistive vessels (arterioles), thus exhibiting cardioprotective effects [22–24].

IMR is not affected by hemodynamic conditions and has good correlation with absolute coronary flow and true microvascular resistance [25], and represents reliable parameter for invasive on-site assessment of microcirculatory function [26]. In fact, Fearon et al. [27] demonstrated that IMR is the strongest predictor for the improvement of left ventricular function, with significant correlation with wall motion score index and peak CK. Finally, it has been demonstrated that high IMR after primary PCI was associated with higher death rate and rehospitalization for heart failure in medium term follow-up [28].

Some previous studies [29–32] have demonstrated that Nicorandil with its cardioprotective effect improves microvascular dysfunction, and might be given intravenously before starting pPCI or as an intracoronary bolus. The impact of Nicorandil in patients with stable angina was demonstrated in IONA (Impact Of Nicorandil in Angina) [33] randomized trial wherein 5126 patients with established coronary heart disease (previous MI, CABG), or a positive exercise test already on the optimal antianginal drug therapy, were included. All patients were divided into 2 groups, treated with Nicorandil or placebo with the follow-up of 1–6 years. In Nicorandil treated group patients had significantly less frequent events like coronary death, non-fatal MI, and unplanned hospital admission. Ono et al. [34], in the study with Nicorandil pretreatment (IV bolus 4 mg) followed by constant infusion at 8 mg/h for 24 h vs. placebo in patients with acute myocardial infarction undergoing pPCI, examined nicorandil effect on reactive oxygen species (ROS) formation by measuring urinary excretion of PGF2α (8-epi-prostaglandin F2α–specific marker for ROS formation) and clinical outcome. Nicorandil pretreatment almost completely inhibited the ROS formation (decrease in urinary excretion of 8-epi-PGFα) resulting in inhibition of no-reflow phenomenon and improvement in LV function vs. control group at 6 month after reperfusion therapy. Ishii et al. [12] performed 5 years follow-up randomized study in patients with STEMI where Nicorandil was given before pPCI as intravenous infusion of 12 mg (in 100 ml of saline) during 20–30 min period. They found that single admission of Nicorandil improved not only early but also late clinical events, including cardiovascular death or hospital admission due to worsening congestive heart failure. Finally, Ito and al. [35] in 60 patients with STEMI undergoing pPCI evaluated the effects of Nicorandil (2 mg intracoronary) and nitroglycerine (250mcg intracoronary) in cross-over fashion on microvascular function as measured by IMR. They have demonstrated that Nicorandil reduced microvascular dysfunction after pPCI more effectively than did nitroglycerine.

Recovery of coronary microvascular function takes place in the first 24–48 h after primary PCI and is a dynamic process. Generally, CFR>2 is taken for the normal cut-off in the most clinical situations. CFR during adenosine induced hyperemia and diastolic deceleration time provide prognostic information to standard clinical and echocardiography data for infarct size prediction and myocardial functional recovery [19, 36].

These data imply that preservation of coronary microcirculatory function after primary PCI in STEMI patients is important for limitation of the infarct size, and improvement of prognosis independently from other factors. Therefore aggressive therapeutic strategy aimed to microcirculation could probably prevent adverse remodeling due to large infarction size. Non-invasively assessed IRA coronary flow reserve and diastolic deceleration time on the first and second day after primary PCI in patients with first STEMI predict final infarct size, independently of other covariates [37].

### Study limitation

This study included rather small number of patients that was estimated on the basis of sample size calculation for IMR improvement in patients with stable coronary artery disease. The follow-up period of 3 months might be short, but the effects on nicorandil in the treatment group clearly demonstrated improvement of IMR, CFR and WMSI. The Nicorandil effects were not compared to the control group or other drugs that might improve microcirculatory function due to limited access and specific package of Nicorandil that precluded proper randomization procedure in patients with STEMI undergoing pPCI. Transthoracic Doppler echocardiography as a technique could not be considered as a substitute for other dedicated techniques that can directly assess transmurality of myocardial viability, such as magnetic resonance imaging, but could be useful in providing additional information about coronary pathophysiology and prognosis, considering the broad availability and low cost of the method [19].

## Conclusion

Intracoronary Nicorandil administration in patients with STEMI undergoing pPCI significantly improved microvasculatory function by decreasing IMR and increasing CFR. Larger randomized studies are needed to confirm these effects particularly in comparison to the other drugs that might improve microcirculatory function.

### Clinical implications

Our group of patients consisted of patients with STEMI undergoing primary PCI with “small to moderate” size myocardial infarction as expressed by CK, wall motion score index, as well as IMR. Even in this group of patients nicorandil produced significant decrease in IMR and improvement in microcirculatory function. Thus, it can be expected that in the subset of patients with large myocardial infarction and real no-reflow phenomenon, nicorandil might induce most beneficial effects on microcirculatory and myocardial function.

## Abbreviations

STEMI: ST-segment elevation myocardial infarction
pPCI: Primary percutaneous coronary intervention
IMR: Index of microcirculatory resistance
CFR: Coronary flow reserve
EF: Ejection fraction
WMSI: Wall motion score index
MI: Myocardial infarction
CABG: Coronary artery bypass grafting
IRA: Infarct related artery

## References

[1] Resnic F, Wainstein M, Lee M, Behrendt D, Wainstein R, Ohno-Machado L (2003). No-reflow is an independent predictor of death and myocardial infarction after percutaneous coronary intervention. Am Heart J.

[2] Porto I, Ashar V, Mitchell A (2006). Pharmacological management of no reflow during percutaneous coronary intervention. Curr Vasc Pharmacol.

[3] Camici P, Crea F (2007). Coronary microvascular dysfunction. N Engl J Med.

[4] Marzilli M, Orsini E, Marraccini P, Testa R (2000). Beneficial effects of intracoronary adenosine as an adjunct to primary angioplasty in acute myocardial infarction. Circulation.

[5] Niccoli G, Rigaztieri S, De Vita MR, Valgimigli M, Corvo P, Fabbiocchi F (2013). Open-label, randomized, placebo controlled evaluation of intracoronary adenosine or nitroprusside after thrombus aspiration during primary percutaneous intervention for the prevention of microvascular obstruction in acute myocardial infarction. J Am Coll Cardiol.

[6] Mahaffey K, Puma J, Barbagelata A, DiCArli M, Leesar M, Brown K (1999). Granger and for the AMISTAD Investigators. J Am Coll Cardiol.

[7] Babbitt D, Virmani R, Vildibill H, Daughtry Norton E, Forman M (1990). Intracoronary adenosine administration during reperfusion following 3 hours of ishemia: effects on infarct size, ventricular function, and regional myocardial blood flow. Am Heart J.

[8] Taniyama Y, Ito H, Iwakura K, Masuyama T, Hori M, Takiuchi S (1997). Beneficial effects of intracoronary verapamil on microvascular and myocardial salvage in patients with acute myocardial infarction. J Am Coll Cardiol.

[9] Grover G, Garlid K (2000). ATP-Sensitive potassium channels: a review of their cardioprotective pharmacology. J Mol Cell Cardiol.

[10] Morin D, Assaly R, Paradis S, Berdaux A (2009). Inhibition of mitochondrial membrane permeability as a putative pharmacological target for cardioprotection. Curr Med Chem.

[11] Lim S, Bae E, Jeong M, Kang D, Lee Y, Kim K (2004). Effect of combined intracoronary adenosine and nicorandil on no-reflow phenomenon during percutaneous coronary intervention. Circulation.

[12] Ishii H, Ichimiya S, Kanashiro M, Amano T, Imai K, Murohara T (2005). Impact of a single intravenous administration of nicorandil before reperfusion in patients with ST-segment elevation myocardial infarction. Circulation.

[13] Kushner G, Hand M, Smith C, King B, Anderson L, Antman M (2009). 2009 focused updates: ACC/AHA guidelines for the management of patients with ST-elevation myocardial infarction (updating the 2004 guideline and 2007 focused update) and ACC/AHA/SCAI guidelines on percutaneous coronary intervention (updating the 2005 guideline and 2007 focused update): a report of the American College of Cardiology Foundation/American Heart Association Task Force on Practice Guidelines. J Am Coll Cardiol.

[14] Brodie BR, Stuckey TD, Hansen C, VerSteeg DS, Muncy DB, Moore S (2005). Relation between electrocardiographic ST-segment resolution and early and late outcomes after primary percutaneous coronary intervention for acute myocardial infarction. Am J Cardiol.

[15] Cannon P, Braunwald E (1996). Time to reperfusion: the critical modulator in thrombolysis and primary angioplasty. J Thromb Thrombolysis.

[16] Henriques J, Zijlstra F, WJ van’Hof A, Jan de Boer M, Dambrink JH, Gosselink M (2003). Angiographic assessment of reperfusion in acute myocardial infarction by myocardial blush grade. Circulation.

[17] Schiller B, Shah M, Crawford M, DeMaria A, Devereux R, Feigenbaum H (1989). Recommendations for quantitation of the left ventricle by two-dimensional echocardiography. J Am Soc Echocardiogr.

[18] Tesic M, Djordjevic-Dikic A, Beleslin B, Trifunovic D, Giga V, Marinkovic J (2013). Regional difference of microcirculation in patients with asymmetric hypertrophic cardiomyopathy: transthoracic Doppler coronary flow velocity reserve analysis. J Am Soc Echocardiogr.

[19] Djordjevic-Dikic A, Beleslin B, Stepanovic J, Giga V, Tesic M, Dobric M (2011). Prediction of myocardial functional recovery by noninvasive evaluation of basal and hyperemic coronary flow in patients with previous myocardial infarction. J Am Soc Echocardiogr.

[20] Wu M, Huang Z, Xie H, Zhou Z (2013). Nicorandil in patients with acute myocardial infarction undergoing primary percutaneous coronary intervention: a systematic review and meta-analysis. PLoS ONE.

[21] Erbel R, Heusch G (2000). Coronary microembolization. J Am Coll Cardiol.

[22] Eeckhout E (2003). Nicorandil: a drug for many purposes: too good to be true?. Eur Heart J.

[23] Kloner R, Rezkalla S (2004). Cardiac protection during acute myocardial infarction: where do we stand in 2004?. J Am Coll Cardiol.

[24] Hirohata A, Yamamoto K, Hirose E, Kobayashi Y, Takafuyi H, Sano F (2014). Nicorandil prevents microvascular dysfunction resulting from PCI in patients with stable angina pectoris: a randomized trial. Eurointervention.

[25] Fearon W, Aarnousde W, Pijls N, de Bruyne B, Balsam L, Cooke D (2004). Microvascular resistance is not influenced by epicardial coronary artery stenosis severity. Experimental validation. Circulation.

[26] Sezer M, Umman B, Okcular I, Nisanci Y, Umman S (2007). Relationship between microvascular resistence and perfusion in patients with reperfused acute myocardial infarction. J Int Cardiol.

[27] Fearon W, Shah M, Ng M, Brinton T, Wilson A, Tremmel J (2008). Predictive value of index of microvasculatory resistance in patients with ST-segment elevation myocardial infarction. J Am Coll Cardiol.

[28] Fearon WF, Low AF, Yong AS, McGeoch R, Berry C, Shah MG (2013). Prognoctis value of the index of microcirculatory resistance measured after primary percutaneous intervention. Circulation.

[29] Iida S, Kinoshita H, Holford N (2008). Population pharmacokinetic and pharmacodynamic modeling of the effects of nicorandil in the treatment of acute heart failure. Br J Clin Pharmacol.

[30] Ito H, Taniyama Y, Iwakura K, Nishikawa N, Masuyama T, Kuzuya T (1999). Intravenous nicorandil can preserve microvascular integrity and myocardial viability in patients with reperfused anterior wall myocardial infarction. J Am Coll Cardiol.

[31] Sugimoto K, Ito H, Iwakura K, Ikushima M, Kato A, Kimura R (2003). Intravenous nicorandil in conjuction with coronary repefusion therapy is associated with better clinical outcome and functional outcomes in patients with acute myocardial infarction. Circulation.

[32] Ishii H, Amano T, Ichimiya S, Matsubara T, Kanashiro M, Murohara T (2006). Effects of intravenous nicorandil before reperfusion for acute myocardial infarction in patients with stress hyperglycemia. Diabetes Care.

[33] The IONA Study Group (2002). Effect of nicorandil on coronary events in patients with stable angina: the Impact of nicorandil in Angina (IONA) randomized trial. Lancet.

[34] Ono H, Osanai T, Ishizaka H, Hanada H, Kamada T, Onodera H (2004). Nicorandil improves cardiac function and clinical outcome in patients with acute myocardial infarction undergoing primary percutaneous coronary intervention: role of inhibitory effect on reactive oxygen species formation. Am Heart J.

[35] Ito N, Nanto S, Doi Y, Kurozumi Y, Natsukawa T, Shibata H (2013). Beneficial effects of intracoronary nicorandil on microvascular dysfunction after primary percutaneous coronary intervention: demonstration of its superiority to nitroglycerin in a cross-over study. Cardiovasc Drugs Ther.

[36] Giga V, Dobric M, Beleslin B, Sobic-Saranovic D, Tesic M, Djordjevic-Dikic A (2013). Estimation of infarct size using transthoracic Doppler echocardiographic measurement of coronary flow reserve in infarct related and reference coronary artery. Int J Cardiol.

[37] Trifunovic D, Sobic-Saranovic D, Beleslin B, Stankovic S, Marinkovic J, Orlic D (2014). Coronary flow of the infarct artery assessed by transthoracic Doppler after primary percutaneous coronary intervention predicts final infarct size. Int J Cardiovasc Imaging.

